# Is Ovulation the Sole Cause of Menstruation?

**Published:** 1874-06-01

**Authors:** 


### IS OVULATION THE SOLE CAUSE OF MENSTRUATION?

DR. C. C. Matteson, in an essay published in the *Obstetrical Journal*, (April, 1874,) reviews the evidence in relation to this question and gives brief abstracts of ten cases, collated from various sources, in which menstruation has continued regular after the removal of the ovaries. In one case reported by Dr. W. L. Atlee, menstruation continued regular for ten years, after the operation, when it ceased, at the age of forty-six years.

In concluding his essay the author says:

"The attention of the medical world has, only for a comparatively short time, been directed to the consideration of this phenomenon, so that the notes of many of the cases are necessarily incomplete. In attempting to account for the few instances, at first reported, the discharge was attributed to habit. It was, and indeed is now, argued, that the catamenia, being established through the agency of ovulation, continued to appear, after the removal of the ovaries, from a habit of the economy. This argument might be worthy of consideration in reference to the earlier and incomplete cases, where the flux appeared but once or twice; but it hardly seems rational to attribute to such an agency as habit, a regular, periodical discharge continuing through nine or ten years. Moreover, were the continuance of the menses due to habit alone, we should justly expect that, as the exciting cause became the more remote, gradually the effect would become less and less marked. Is this the fact in the cases recorded? Apparently not so. Indeed, Dr. Battey states that the metrostaxis was 'more profuse and hemorrhagic' than ordinary menstruation. Under these circumstances, therefore, habit seems insufficient to account for the phenomenon presented. What then remains? Can we fail to admit, with our present knowledge, that menstruation does take place when the ovaries are wanting?

Attacks have been made upon the ovular theory from various quarters, and none of these assaults have been able to overthrow it. Yet, among its strong defences was ranked the invariable cessation of menstruation upon the removal of the ovaries, but this defence seems hardly tenable, under our present information. Future investigations will lead into new trains of thought, and prove or disprove all previous theories; but from the facts presented, and arguments entered into, it seems but right to admit that ovulation cannot be the *sole* cause of menstruation.

ILLINOIS STATE MEDICAL SOCIETY.—The Annual Session of this Society convened in this city on Tuesday, May 19th. There was a large attendance of members and delegates from all parts of the State, and an unusual degree of interest was maintained throughout the meeting.

On Wednesday evening the guests from abroad were entertained by the Profession of the city at a banquet at the Grand Pacific Hotel.

The dinner was served in the most elegant and elaborate style of this truly grand and magnificent hotel, and the evening proved a most happy and enjoyable one to all present.

The first toast to *Our Guests* was very happily and appropriately responded to by Prof. J. Adams Allen, and was followed by a reply from Dr. T. F. Worrell, of Bloomington, ex-President of the Society. Sentiments were also briefly responded to, on behalf of the Chicago Medical Society, by Dr. W. E. Quine; on behalf of the Chicago Society of Physicians and Surgeons, by Dr. John Bartlett, and on behalf of the Legal Profession, by Judge H. Booth.

The farewell *good night* sentiment was replied to by Prof. N. S. Davis.

As the artificially *enlivening* and *stimulating* influences of the *ruby wine* or the *sparkling champagne* were not called into requisition, the universally pervading spirit of mirth and jollity, progressively increasing to an uproarious pitch at the close, could only be explained on the supposition that over-eating, as well as an excess in drinking, may be capable of overpowering or intoxicating the intellectual faculties.

On Thursday afternoon the members visited, by invitation, the Rush Medical College and Cook County Hospital, and the Chicago Medical College and Mercy Hospital. At the former institution an experiment in the transfusion of blood in a dog was exhibited by Prof. J. W. Freer, and a practical exhibition of the use of the aspirator given by Prof. E. Powell.

The officers elected by the Society for the ensuing year were :

*President*—Prof. J. H. Hollister.

*Treasurer*—Dr. W. E. Quine.

*Permanent Secretary*—Dr. T. D. Fitch.

The next place of meeting to be held at Jacksonville.

We had hoped to be able to give a detailed account of the proceedings of the Society, the discussions, etc., in this number of THE EXAMINER, but are obliged to defer it until our next issue.

---

IN a recent number of the *Berlin Klin., Wochensch*, Dr. Fr. Schultze calls attention to a few cases of tetanus that are highly interesting in an etiological point of view. These three cases very strangely supervene upon severe febrile attacks, though the Dr. does not state the exact period in the course of the disease at which the symptoms of tetanus commenced to appear. In the first case it was evolved from an attack of small-pox without any other known predisposing cause, while in the other two cases it was from typhoid fever.

